# Reliability of Physiological Responses Induced by Basic Emotions: A Pilot Study

**DOI:** 10.1186/s40101-019-0209-y

**Published:** 2019-11-28

**Authors:** Eun-Hye Jang, Sangwon Byun, Mi-Sook Park, Jin-Hun Sohn

**Affiliations:** 1 Welfare & Medical ICT Research Department, Electronics and Telecommunications Research Institute (ETRI), 218 Gajeong-ro, Yuseong-gu, Daejeon, 34129 Republic of Korea; 2 Department of Electronics Engineering, Incheon National University, 119 Academy-ro, Yeonsu-gu, Incheon, Republic of Korea; 3 Department of Rehabilitation Counselling, Seoul Hanyoung University, 290-42 Kyoungin-ro, Guro-gu, Seoul 08274 Republic of Korea; 4 Department of Psychology, Brain Research Institute, Chungnam National University, 99 Daehak-ro, Yuseong-gu, Daejeon, 34134 Republic of Korea

**Keywords:** Emotion, Physiological responses, Consistency, Reliability, Autonomic nervous system

## Abstract

**Background:**

Although emotion-specific autonomic responses based on the discrete theory of emotion have been widely studied, studies on the reliability of physiological responses to emotional stimuli are limited. In this study, we aimed to assess the reliability of physiological changes induced by the six basic emotions (happiness, sadness, anger, fear, disgust, and surprise) that were measured during 10 weekly repeated experiments.

**Methods:**

Twelve college students participated, and in each experiment, physiological signals were collected before and while participants were watching emotion-provoking film clips. Additionally, the participants self-evaluated the emotions that they experienced during the film presentation at the end of each emotional stimulus. To avoid adaptation of participants to identical stimuli during repeated measurements, we used 10 different film clips for each emotion, and thus a total of 60 film clips over 10 weeks were used. Physiological features, such as skin conductance level (SCL), fingertip temperature (FT), heart rate (HR), and blood volume pulse (BVP), were extracted from the physiological signals. Two reliability indices, Cronbach’s alpha and intraclass correlation coefficient, were calculated from the physiological features to assess internal consistency and interrater reliability, respectively.

**Results:**

We found that SCL, HR, and BVP measured during the emotion-provoking phase over the 10 weekly sessions were more reliable than those assessed at baseline. Furthermore, SCL, HR, and BVP from the emotion-provoking phase exhibited excellent internal consistency and interrater reliability.

**Conclusions:**

Our findings suggest that these features can be used as reliable physiological indices in emotion studies. The results also support the significance of physiological signals as meaningful indicators for emotion recognition in HCI (human computer interface) area.

## Background

Physiological and behavioral changes are fundamental aspects of emotions, and emotions therefore cannot be simply interpreted as “feelings” or mental states [1]. Autonomic responses in emotions have been an active research topic since Cannon [2] first reported the physiology of emotions [3, 4]. Autonomic nervous system (ANS) activity has been viewed as a major component of emotion response in many recent theories of emotion [4]. Cacioppo and colleagues [5, 6] reported reliable differences in autonomic responses between specific emotions, that is, autonomic emotion specificity. They also noted context-specific effects of ANS activity in emotion, which were related to different induction paradigms [5, 6]. Furthermore, ANS responses exhibited more consistent valence-specific patterns than emotion-specific patterns: negative emotions were accompanied by stronger autonomic responses than positive emotions [4].

As emotion-specific ANS responses have been widely studied (see Kreibig’s review article [4]), previous studies have attempted to assess the reliability of physiological responses induced by basic emotions [7]. For example, the temporal stability of physiological responses [8–13] and the reliability of event-related desynchronization in EEGs [14, 15] have been studied. Typically, intervals of 2–4 weeks were introduced between test-retest experiments to measure the stability and consistency of physiological responses [16–19]. Various other biomarkers, including blinking responses, respiratory sinus arrhythmia, heartbeat period, salivary cortisol, and startle response, have also been tested [16–19]. However, most of these studies relied on a single repeat (test-retest) and used an identical stimulus for emotion elicitation, which may complicate the interpretation of results. Repeated measurements with the same stimulus may be affected by adaptation of participants to stimuli or learning effects (e.g., habituation). Furthermore, consistency over a relatively long period has not been evaluated.

In most studies on autonomic specificity of basic emotions, the direction of changes in ANS activity was evaluated as the difference between the baseline measurement and emotional condition [4], in which the baseline activity was used as a reference for observing state change in psychophysiological responses [20]. Since the quality of the baseline data against which autonomic change is evaluated relies on the methodology, previous studies have developed baseline calibration techniques for establishing an appropriate baseline, which would enable reliable assessment of psychophysiological changes during emotional state [21]. However, intra- and interindividual variations in the baseline level have not been studied separately. In particular, intraindividual variations in the baseline have been implicitly assumed to be not substantial.

Therefore, we aimed to examine the consistency of physiological responses that are measured multiple times over a relatively long period, using non-identical stimuli. We evaluated skin conductance level (SCL) and heart rate (HR), which are the most frequently reported measures of autonomic function [14]. We also measured fingertip temperature (FT) [22] and blood volume pulse (BVP) [23, 24] as additional indicators of the ANS activity. Based on these physiological measurements, we evaluated the consistency of baseline activity and autonomic responses related to six basic emotions (happiness, sadness, anger, fear, disgust, and surprise) during 10 weekly sessions. We hypothesized that physiological responses would show high consistency in both the baseline and emotional phases.

## Methods

### Participants

Twelve healthy subjects between 19 and 23 years of age (6 males and 6 females, mean age ± SD, 21.0 ± 1.98) participated in the 10-week longitudinal study. None of the participants reported any history of psychiatric, neurological, or pulmonary disorders, or use of medication that would affect the peripheral or central nervous system. All participants provided written informed consent before the experiment began and received US$20 per session as a reward for their participation. The study was approved by the Institutional Review Committee of Chungnam National University (No. 201309-SB-004-01).

### Emotion-provoking stimuli

We used film clips as emotional stimuli to provoke six basic emotions, since they are among the most effective stimuli to elicit affective responses in an experimental laboratory [25–28]. Film stimuli are dynamic media that provide visual and auditory input simultaneously, which contain more information relevant to the development of integrated and sustained emotional responses compared with still pictures (e.g., International Affective Picture System; IAPS), which elicit only short-lived affective responses [25–28]. Therefore, presenting film stimuli is an ecologically valid methodology to induce different affective states [25–28]. Additionally, they can evoke patterned psychophysiological responses, which may help to identify changes in the ANS during emotion elicitation [28].

A total of 60 film clips (10 clips per emotion) were excerpted from a variety of movies and TV programs, including documentaries and dramas (Table 1). Each clip was 2–4-min long. These film clips were used in our previous study and effectively induced the intended emotions in participants [29]. The stimuli were counterbalanced to minimize order effects.

**Table 1:** Description of the emotion-provoking film clips

Emotion	Description of contents
Happiness	Joyful scenes showing victories, weddings, or laughing
Sadness	Frustration or grief scenes showing death of a parent or partner
Anger	Deliberately harmful and unfair behaviors, such as a massacre or violent beating
Fear	Scary scenes showing a ghost or haunted house
Disgust	Disgusting scenes showing mutilation, butchery, or contamination
Surprise	Sudden or unexpected screaming scenes caused by a startling accident

### Psychological measurements

Psychological responses of each participant were assessed by a self-rating questionnaire used in our previous study [29]. This includes three questions. First, participants were asked to label what specific emotion they had experienced during exposure to each stimulus. They selected one of seven choices: happiness, sadness, anger, fear, disgust, surprise, and not applicable. Second, participants evaluated the emotion’s intensity, which reflected how strongly they felt the emotion during presentation of the stimulus, using an 11-point Likert scale, ranging from 1 (weakest) to 11 (strongest) [30]. Finally, the participants described the scene in which they felt the strongest emotion during exposure to each stimulus.

**Fig. 1:**
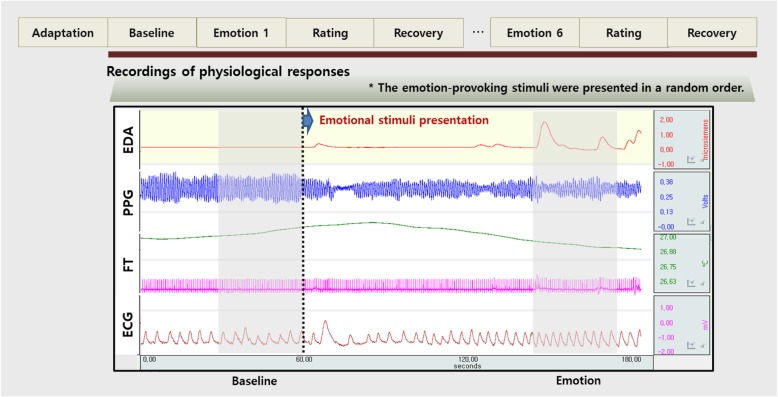
Experimental procedure for physiological data acquisition

We used the results from the first and second questions as psychological responses. The appropriateness of the stimuli was evaluated by the first question, which reflects consistency between the intended emotion of the stimuli and the emotion experienced by the participants. This was assessed as the percentage of the participants who labeled the intended emotion correctly. The effectiveness of the stimuli was evaluated by the second question, which represents the intensity of emotion experienced by the participants for each stimulus. This used raw scores from the participants’ ratings of each emotion’s intensity. The third question was used to determine the interval in the emotion-provoking phase, in which physiological data were analyzed (Fig. 1).

### Procedure

The experiment was conducted in a soundproof room, which reduced noise by at least 35 dB, to block outside noises or artifacts. Participants sat on a comfortable chair placed 2 m away from a 38-in TV monitor located in the center of the room. Prior to the experiment, electrodes for acquiring physiological signals were placed on participants’ wrists, fingers, and ankle. Alcohol swabs and cotton pads were used to clean skin surfaces before attachment. The measurement began with a 60-s baseline phase, during which physiological signals were recorded and without any emotional stimulus (a blue screen presented without an auditory stimulus). Then, participants were presented with an emotion-provoking stimulus for 2–4 min. After the film clip presentation, they were asked to complete the three questions described above [28]. After the ratings, they were given 2 min to allow recovery of their emotional state. Then, the measurement started with a new baseline again. This cycle of baseline phase, emotion-provoking phase, self-ratings, and recovery was repeated six times for each emotion. The order of stimuli was randomized for each participant. Total length of the experiment was ~ 1.5 h. Previous studies on emotion-specific ANS responses have used a separate neutral stimulus as a control condition. However, baseline measurement without any stimulus has also been used when a neutral stimulus is not favorable. We decided not to use a neutral film as a control since presenting a film clip itself can induce undesirable emotion elicitation in participants. Instead, we used a blue screen without any auditory stimulus during the baseline measurement.

The same experimental procedure was repeated 10 times on a weekly basis for each participant. To control the factors that could affect the baseline physiological responses, we contacted the participants by telephone a day before the scheduled experiment and instructed them to avoid smoking and consuming alcohol and caffeine after 8 pm. They were also asked to get sufficient sleep. Furthermore, the experiment was repeated over 10 weeks at the same time during working hours (10 am–3 pm) and on the same day of the week for each participant. We changed film clips used for each emotion every week to avoid habituating participants by repeating the same stimulus. Therefore, 10 different film clips were prepared for each emotion, and a total of 60 film clips were used for 10 weeks.

### Physiological measurement

We used the MP100WS system (Biopac Systems Inc., USA) to measure electrodermal activity (EDA), electrocardiogram (ECG), fingertip temperature (FT), and blood pulse volume (BVP). Display of recorded signals and data analysis were performed with AcqKnowledge software version 3.9.1 (Biopac Systems Inc., USA). An example of physiological data acquired before and during a fearful stimulus is shown in Fig. 1.

The EDA signal was recorded using an EDA100C amplifier, which applies a constant voltage (0.5 V) between two electrodes to measure skin conductance. The amplifier was connected to a set of TSD203 electrodes, which were attached to the palmar surfaces of the middle phalanges of the first and second fingers of the non-dominant hand. The electrodes were filled with Electrode Paste EC33 (Grass Technologies, USA). Sampling rate was 200 Hz. After movement and electrode contact artifacts removed, skin conductance level (SCL, in μS) was calculated by averaging skin conductance signals for a 30-s interval that was determined by the self-rating questionnaire.

The ECG signal was recorded using an ECG100 amplifier, LEAD110S electrode leads, and EL503 electrodes. A lead I configuration was used for ECG measurement, in which three electrodes were attached to the bilateral wrists and left ankle. Sampling rate was 200 Hz. Heart rate (HR, in beats per minute) was analyzed using AcqKnowledge, which detected R-waves in the ECG signals and calculated consecutive R–R intervals. Finally, the mean HR (meanHR) was calculated by averaging HR values for the 30-s interval.

The FT signal was recorded using an FT100C amplifier and TSD202 temperature probe. The probe was attached to the volar surface of the distal phalanx of the little finger of the non-dominant hand. The mean FT (meanFT) was calculated by averaging FT values for the 30-s interval.

The BVP signal was recorded using a PPG100C amplifier and TSD200 transducer, which measured infrared reflectance of varying blood flow from the volar surface of the distal phalanx of the thumb of the non-dominant hand. Sampling rate was 200 Hz. The mean BVP (meanBVP) was calculated by averaging BVP signals for the 30-s interval.

As a result, total four physiological features, SCL, meanHR, meanFT, and meanBVP, were extracted from the measurement and used for data analysis.

### Data analysis

All statistical analyses were performed using SPSS software version 18.0 (SPSS-IBM, Chicago, IL, USA). As psychological responses, the appropriateness and effectiveness of each emotion were evaluated by averaging 12 participants’ ratings. To analyze physiological signals, a 30-s interval was selected from the total length for the baseline and each emotion-provoking phase. For the baseline, the final 30 s was used [29]. For the emotion-provoking phase, the interval was selected based on the participants’ ratings. The participants described the scene in which they felt the strongest emotion during exposure to each stimulus. For example, a participant reported that she felt disgusted while watching the scene of a man putting his hand inside a dirty toilet, and we analyzed the 30-s interval (26–55 s) containing this scene. Physiological features during this interval were evaluated as described above. We excluded the physiological data from participants who failed to elicit the targeted emotion during the analysis.

We adopted the most frequently used method for the measurement and analysis of emotion-specific ANS response. In particular, in most previous studies, the 30- or 60-s interval immediately before the stimulus presentation has been selected as a baseline phase [4]. Other common intervals are 1/2-, 10-, 120-, 180-, and 300-s intervals. In addition, the averaging period did not influence the reported pattern of physiological responses [4], suggesting that a 30-s interval before the start of the stimulus can be considered as an appropriate baseline measurement. However, we cannot completely exclude the effect of anticipation of the stimuli on variations during the baseline.

We assessed the internal reliability of physiological responses observed during the 10 sessions, using Cronbach’s alpha and intraclass correlation coefficient (ICC). Cronbach’s alpha is widely used to evaluate internal consistency [31], which is defined as

$$ \upalpha =\frac{k}{k-1}\ \left(1-\frac{\sum {s}_i^2}{s_T^2}\right), $$

where *k* is the number of sessions, ${s}_i^2$ is the variance of the *i*th session, and ${s}_T^2$ is the variance of the total score formed by summing all the sessions. Furthermore, ICC is a widely used reliability index in test-retest and interrater reliability analyses [32–34], which assesses how strongly units in the same group resemble each other. The ICC coefficient is defined as

$$ \rho =\frac{{\mathrm{MS}}_{\mathrm{R}}-{\mathrm{MS}}_{\mathrm{E}}}{{\mathrm{MS}}_{\mathrm{R}}+\left(k-1\right){\mathrm{MS}}_{\mathrm{E}}}, $$

where MS_R_ is mean square for rows of raters or measurements, MS_E_ is mean square for error, and *k* is number of raters or measurements. ICC estimates and their 95% confidence intervals were calculated based on the model of single rater/measurement, consistency, and 2-way mixed-effects. Reliability indices less than 0.50 are indicative of poor reliability, values between 0.50 and 0.75 indicate moderate reliability, values between 0.75 and 0.90 indicate good reliability, and values greater than 0.90 indicate excellent reliability [32].

We compared physiological responses between the baseline and emotion-provoking phases using the Wilcoxon test since the Shapiro-Wilk test revealed that physiological features were not normally distributed. For this statistical test, we used all data (10 trials × 12 subjects), except for data from participants who failed to elicit the targeted emotion, which were excluded in the calculation. Therefore, the amount of data used in the analysis was not identical for all conditions. For example, in the happiness condition, we evaluated data from 11 subjects for the eighth and tenth sessions because one subject failed to report the intended emotion during these sessions. For the remainder of the sessions, data from all 12 subjects were included in the calculation.

## Results

### Validity of psychological responses

Psychological responses to emotional stimuli were assessed by their appropriateness and effectiveness (Tables 2 and 3). The mean appropriateness of each emotion ranged from 75 to 100%, and the overall mean appropriateness from all the sessions and emotions was 93.3% (Table 2). These results suggest that the film clips were valid stimuli for provoking the intended emotions. The mean appropriateness was higher in happiness, sadness, and disgust, compared with anger, fear, and surprise. The mean effectiveness of each emotion ranged from 8.4 to 10.8 points, and the overall mean effectiveness from all the sessions and emotions was 9.4 points (Table 3). These results also suggest that our stimuli effectively provoked strong emotions.

**Table 2:** Mean appropriateness from participants’ ratings

Session	Emotion						Mean (%)
	HAP (%)	SAD (%)	ANG (%)	FEA (%)	DIS (%)	SUR (%)	
1	100	92	75	75	75	75	83
2	100	100	75	100	92	92	94
3	100	100	75	83	92	100	93
4	100	100	75	92	100	100	95
5	100	100	92	92	92	83	94
6	100	100	92	92	100	83	95
7	100	75	92	83	100	100	92
8	92	100	83	100	83	83	92
9	100	100	92	100	100	83	96
10	92	100	92	75	100	75	91
Mean (%)	98	96	84	89	94	89	93.3

*HAP*, happiness; *SAD*, sadness; *ANG*, anger; *FEA*, fear; *DIS*, disgust; *SUR*, surprise

Appropriateness was assessed as the percentage of the participants who labeled the intended emotion correctly

**Table 3:** Mean effectiveness from participants’ ratings

Session	Emotion						Mean
	HAP	SAD	ANG	FEA	DIS	SUR	
1	8.4 ± 1.07	9.5 ± 1.36	9.7 ± 1.88	10 ± 0.85	10.2 ± 0.95	9.3 ± 1.55	9.5 ± 1.28
2	8.9 ± 1.30	9.1 ± 1.50	9.9 ± 1.07	9.9 ± 1.31	10.8 ± 1.08	9.7 ± 1.38	9.6 ± 1.27
3	8.8 ± 1.31	8.7 ± 1.38	9.7 ± 1.54	9.8 ± 1.71	9.9 ± 1.34	9.7 ± 1.35	9.3 ± 1.44
4	9.6 ± 1.17	9.7 ± 1.08	9.5 ± 1.16	9.6 ± 1.61	10.4 ± 1.04	9.9 ± 1.44	9.7 ± 1.25
5	9.6 ± 1.24	9.3 ± 1.07	9.8 ± 1.68	9.7 ± 1.82	9.7 ± 1.27	9.6 ± 1.50	9.6 ± 1.43
6	9.3 ± 1.49	9.3 ± 0.95	9.4 ± 1.47	9.7 ± 1.08	10.3 ± 0.93	9.6 ± 1.29	9.5 ± 1.20
7	9.3 ± 1.23	8.9 ± 1.48	8.9 ± 1.31	9.6 ± 1.24	9.3 ± 1.31	9.5 ± 1.16	9.3 ± 1.29
8	8.4 ± 1.34	9.1 ± 0.90	9.2 ± 1.07	9.3 ± 1.51	10.2 ± 0.97	9.4 ± 1.38	9.2 ± 1.20
9	9.7 ± 1.51	9.2 ± 1.62	9.5 ± 1.82	9.3 ± 1.08	10.1 ± 1.21	8.6 ± 1.54	9.4 ± 1.46
10	8.8 ± 1.42	9.3 ± 1.48	9.7 ± 1.07	8.7 ± 1.44	10.1 ± 1.16	10.3 ± 0.96	9.5 ± 1.26
Mean	9.1 ± 1.31	9.2 ± 1.28	9.5 ± 1.41	9.6 ± 1.37	10.1 ± 1.13	9.5 ± 1.36	9.4 ± 1.31

*HAP*, happiness; *SAD*, sadness; *ANG*, anger; *FEA*, fear; *DIS*, disgust; *SUR*, surprise

### Reliability of psychological responses

We analyzed reliability of physiological responses to emotional stimuli using Cronbach’s alpha and ICC. Reliability indices for the six emotions are summarized in Table 4 (Additional file 1: Tables S1–S6), which also present the descriptive statistics (mean and standard deviation) of physiological responses during the baseline and emotion-provoking phases. Cronbach’s alphas from baseline measurement ranged from 0.13 to 0.79, and those from emotion-provoking phases ranged from 0.39 to 0.96. Interestingly, when Cronbach’s alphas from the baseline and emotion-provoking phases of the same physiological feature and same emotional stimulus were compared, Cronbach’s alpha was always higher in the emotion-provoking phase than in the baseline, except meanFT during happiness. ICC values from the baseline measurements ranged from 0.10 to 0.70, and those from emotion-provoking phases ranged from 0.38 to 0.96. Similarly, ICC was always higher in the emotion-provoking phase than in the baseline of the same physiological feature and same emotional stimulus, except meanFT during happiness emotion. In particular, Cronbach’s alpha and ICC of SCL, meanHR, and meanBVP from emotion-provoking phases were higher than 0.90 for all emotional conditions.

**Table 4:** Physiological responses and their reliability indices from the baseline and emotion-provoking phases of all emotional conditions

Emotion		SCL (μS)		meanFT (°C)		meanHR (bpm)		meanBVP (V)	
		Baseline	Emotion	Baseline	Emotion	Baseline	Emotion	Baseline	Emotion
HAP	Mean	4.3 ± 2.86	3.9 ± 3.21	34.0 ± 0.36	33.9 ± 0.38	74.9 ± 10.68	74.1 ± 11.72	8.4 ± 0.70	8.5 ± 0.72
	α	.61	.95	.69	.69	.44	.92	.63	.95
	*ρ*	.59.	.95	.69	.69	.38	.91	.67	.95
SAD	Mean	4.4 ± 2.71	4.1 ± 2.79	33.9 ± 0.51	34.0 ± 0.56	73.0 ± 10.18	71.6 ± 10.88	8.3 ± 0.83	8.3 ± 0.88
	α	.63	.95	.36	.54	.48	.96	.57	.96
	*ρ*	.54	.94	.35	.54	.44	.96	.63	.96
ANG	Mean	4.2 ± 2.81	4.1 ± 2.88	33.9 ± 0.46	34.0 ± 0.47	73.3 ± 10.53	71.4 ± 11.05	8.3 ± 0.84	8.4 ± 0.83
	α	.47	.96	.57	.61	.26	.96	.41	.96
	*ρ*	.40	.96	.56	.60	.10	.95	.45	.96
FEA	Mean	4.6 ± 2.80	5.4 ± 2.68	34.0 ± 0.49	34.0 ± 0.48	73.3 ± 10.46	71.9 ± 10.87	8.5 ± 0.92	8.3 ± 0.86
	α	.34	.91	.52	.62	.50	.94	.56	.96
	*ρ*	.30	.91	.52	.61	.40	.94	.56	.96
DIS	Mean	4.1 ± 2.58	4.9 ± 2.99	34.0 ± 0.42	33.9 ± 0.45	74.1 ± 9.74	71.6 ± 10.54	8.4 ± 0.84	8.3 ± 0.82
	α	.57	.94	.13	.39	.46	.96	.71	.96
	*ρ*	.57	.93	.13	.38	.40	.96	.70	.96
SUR	Mean	4.1 ± 2.74	4.9 ± 2.89	34.1 ± 0.40	34.0 ± 0.41	74.0 ± 10.66	71.7 ± 10.66	8.5 ± 0.88	8.3 ± 0.84
	α	.79	.92	.63	.72	.54	.96	.71	.96
	*ρ*	.45	.92	.63	.72	.47	.95	.70	.96

*HAP*, happiness; *SAD*, sadness; *ANG*, anger; *FEA*, fear; *DIS*, disgust; *SUR*, surprise; *α*, Cronbach’s alpha; *ρ*, intraclass correlation

**Fig. 2:**
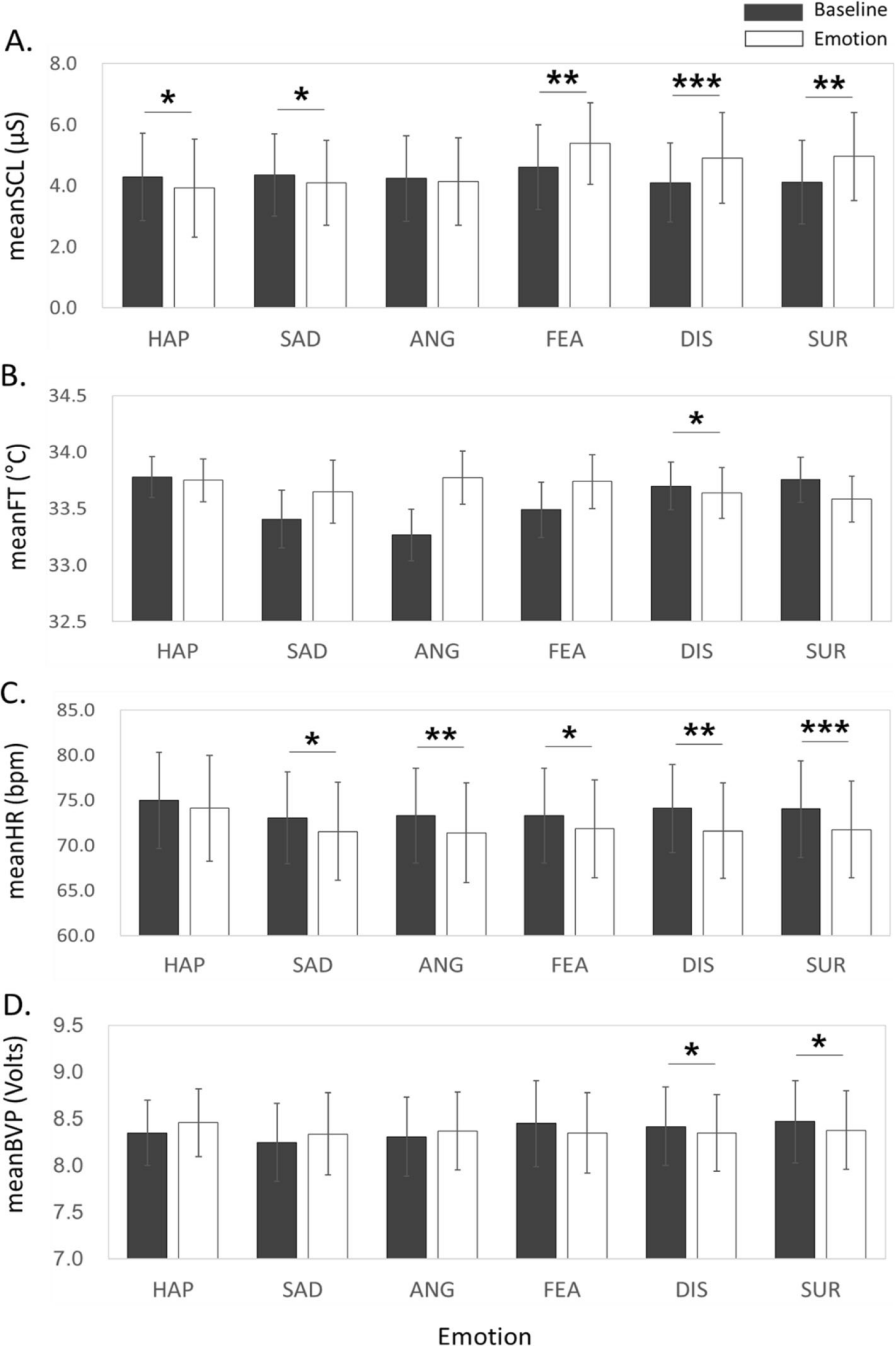
Differences in physiological features between the baseline and six emotional conditions (* *p* < .05, ** *p* < .01, *** *p* < .001, Wilcoxon test). **a** SCL, **b** meanFT, **c** meanHR, **d** meanBVP

Figure 2 shows group level comparisons of physiological features between the baseline and emotion-provoking phases. There were significant differences in the SCL between the two phases in all emotion conditions except for anger; the SCL significantly decreased in the happiness and sad conditions but increased in the fear, disgust, and surprise conditions. The meanFT significantly decreased compared with the baseline during disgust emotion. The meanHR significantly decreased during all emotion-provoking phases, except for happiness, compared with the baseline. The meanBVP significantly decreased during disgust and surprise emotions compared with the baseline.

**Fig. 3:**
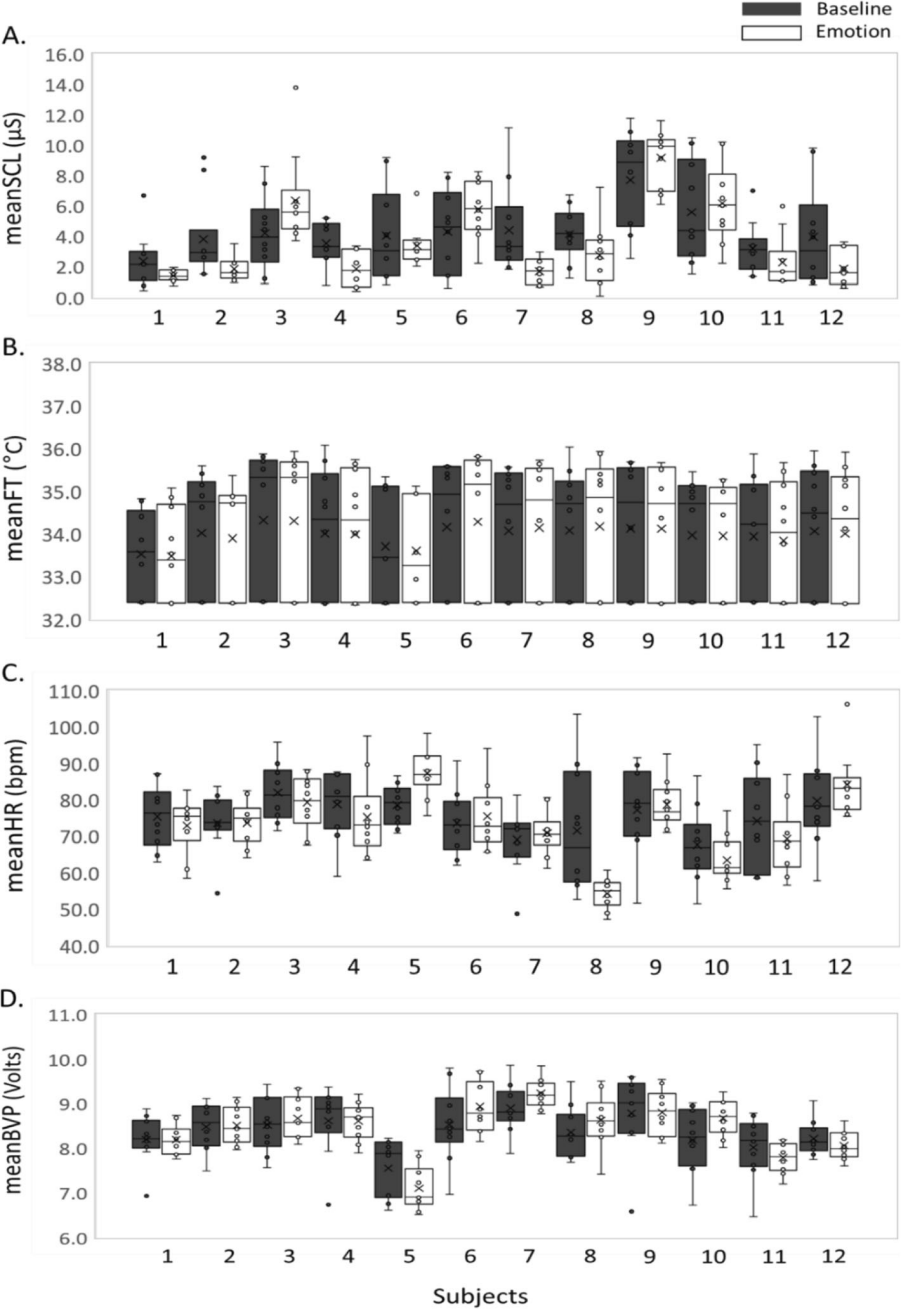
Physiological features acquired from a single emotional condition (happiness) during 10 weekly sessions. Distributions of the features from the baseline and emotion-provoking phases of each subject are compared by the box plots. **a** SCL, **b** meanFT, **c** meanHR, **d** meanBVP

**Fig. 4:**
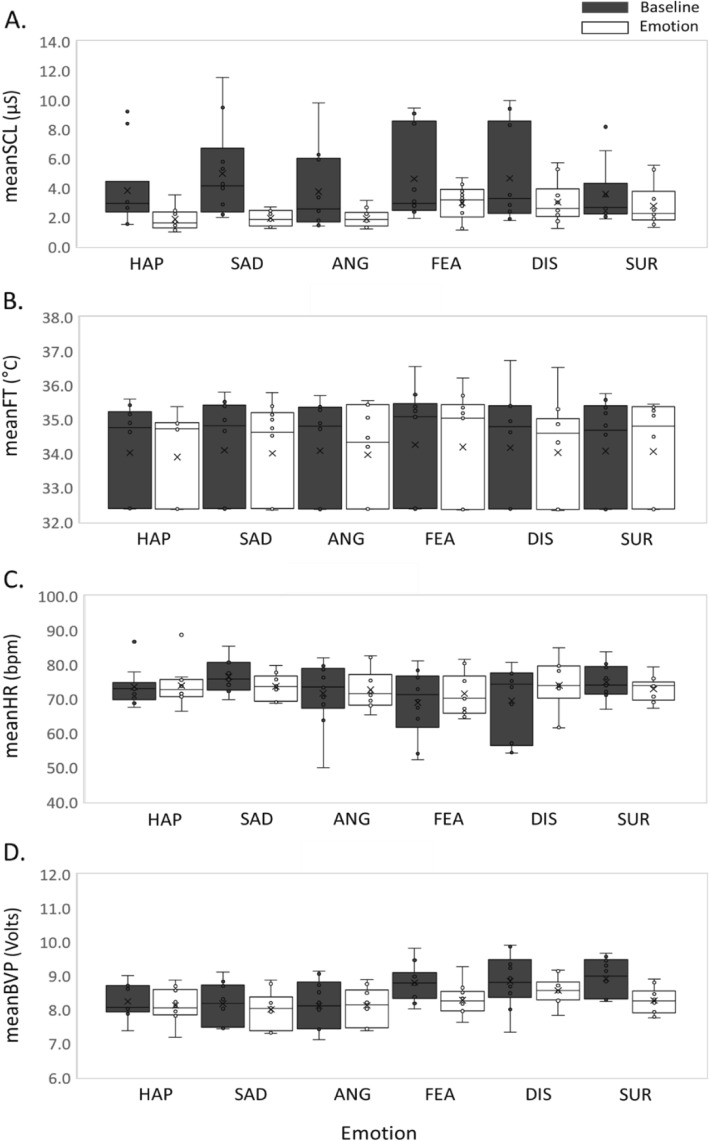
Physiological features acquired from a single subject during 10 weekly sessions. Distributions of the features from the baseline and emotion-provoking phases of each emotional condition are compared by the box plots. **a** SCL, **b** meanFT, **c** meanHR, **d** meanBVP

Figure 3 shows physiological features acquired from a single emotional condition (happiness) during 10 weekly sessions. Box plots in Fig. 3 compare the distributions of the features from the baseline and emotion-provoking phases of each subject. For the happiness condition, most subjects showed larger variations in the baseline than the emotion-provoking phase, except for the meanFT. As an example, Fig. 4 shows physiological features acquired from a single subject during 10 weekly sessions. Box plots in Fig. 4 compare the distributions of the features from the baseline and emotion-provoking phases of each emotional condition. For all six emotions, this subject showed larger variations in the baseline than the emotion-provoking phase, except for the meanFT.

## Discussion

We investigated the reliability of physiological responses induced by emotional stimuli for provoking basic emotions during 10 weekly sessions. The film clips used as emotional stimuli in the present study were confirmed to be appropriate and effective in inducing the intended emotions regardless of the variations in film clips (Tables 2 and 3). Film clips can produce more active and vivid emotional responses than static emotional stimuli (e.g., facial expressions, slides, and imagery) and can be considered advantageous in terms of ecological validity, to the extent that emotions are induced by dynamic visual and auditory stimuli [35, 36].

However, film clips may also evoke other unanticipated emotions. For example, the mean appropriateness of anger, fear, disgust, and surprise stimuli for the first session was 75% (Table 2), indicating that some participants did not select the intended emotions from these stimuli as their experienced emotions. In fact, these results may support Levenson’s [37] claim that emotions are far more complex in that people do not experience only one emotion during a particular situation; instead, they experience several mixed emotions that have dominance over one another in a relatively consistent pattern. Christie and Friedman [36] have applied pattern classification analyses to discriminate seven distinct emotions experienced by subjects based on an 18-item affect self-report scale. In this study, the sadness condition was predicted correctly for 27 out of 34 subjects, whereas the remaining seven subjects were classified as experiencing the amusement, anger, contentment, and disgust conditions [36]. Similarly, when disgust was expected, 15 out of 34 subjects were classified as experiencing amusement, anger, fear, and sadness [36]. These results suggest that it is difficult to elicit a single discrete emotion using any given stimulus, yet it is possible to induce most commonly experienced emotions. Therefore, it may be realistic to aim to acquire the most frequently reported emotion from a given stimulus rather than to expect a perfect match between the intended and experienced emotions. This may partially explain why we observed large variations in individual responses.

We observed that the emotional stimuli significantly affected SCL and meanHR, which are the most frequently reported measures for sympathetic and parasympathetic autonomic activity, suggesting that these two features were indeed strong indicators of ANS responses (Fig. 2). Our results were also consistent with previous studies on physiological responses to emotional stimuli; for example, all emotions, except for contentment, non-crying sadness, and acute sadness, induced an increase in SCL; facial expressions of anger induced decreases in HR and SCL; HR decreased during contentment, non-crying and acute sadness, imminent-threat fear, and mutilation-related disgust conditions [4].

Both reliability indices, Cronbach’s alpha and ICC, were lower in the baseline than in the emotion-provoking phase of all emotions. In particular, Cronbach’s alpha and ICC in the baseline phase ranged from 0.13 to 0.79 and from 0.10 to 0.70, respectively, indicating poor or moderate reliability. These results suggest that intra- and interindividual variations in the baseline level were relatively large. Since an individual’s initial baseline level will affect the degree to which his/her autonomic system responds to emotional stimuli, it is important to assess the baseline and incorporate this information for interpreting physiological changes in response to the emotional stimulus [38]. For example, the differential values between the baseline and emotional states can be used for statistical tests or the baseline can be used as a covariate in analysis of covariance for physiological response during the emotional state.

In contrast, Cronbach’s alpha and ICC of SCL, meanHR, and meanBVP in the emotion-provoking phase both ranged from 0.91 to 0.96, indicating excellent reliability [32]. Therefore, SCL, meanHR, and meanBVP measured from the emotion-provoking phase exhibited excellent internal consistency and reliability throughout the 10 weekly sessions [39, 40]. However, the reliability indices of meanFT during the emotion-provoking phase ranged from 0.38 to 0.72, indicating poor or moderate reliability. In particular, meanFT in disgust showed the lowest reliability (Tables 4 and Additional file 1: Table S5). Similarly, the change in FT in response to disgust stimuli is known to be inconsistent [4]. For example, disgust elicited in relation to mutilation induced consistent increases in HR and SCL but mixed results in FT [4]. Furthermore, the FT response depended on the type of disgust stimuli; FT was unaffected by personalized recall [41], increased by directed facial action or personalized recall [42–44], and decreased by picture or film clip presentations [45–48]. In our previous study [29], the same film stimuli for disgust used in the present study exhibited substantially high appropriateness and effectiveness, and we expected that the disgust stimuli would lead to reliable physiological responses in subjects. Nonetheless, the meanFT during the emotion-provoking phase was not consistent throughout the 10 sessions.

## Limitations

We used 10 different film clips for each emotion to avoid adaptation of participants caused by repeating the same stimulus. However, different contexts designed to elicit the same emotion can lead to different physiological responses. For example, disgust related to contamination and pollution is accompanied by sympathetic-parasympathetic co-activation, whereas disgust related to mutilation, injury, and blood is accompanied by sympathetic deactivation and unchanged vagal activation [4]. We did not investigate the effect of different contexts of the film stimuli for the same targeted emotion on physiological responses. Therefore, to evaluate the reliability more accurately, we may need to subdivide the film stimuli with respect to their contexts.

During the baseline measurement, we did not use a neutral film stimulus because presenting a film clip itself can induce undesirable emotion elicitation. Instead, we used a blue screen without any auditory stimulus. In a future study, we may need to include neutral film clips to evaluate the reliability of physiological responses to the neutral condition.

Participants were given 2 min to allow recovery between emotional conditions. However, a 2-min recovery period may not be sufficient to fully recover from emotional states, especially after a negative emotion inducement (e.g., anger). This may partially explain why we observed large variations in baseline measurement.

## Conclusions

In conclusion, we evaluated the inter- and intraindividual reliability of physiological responses induced by emotional stimuli during 10 weekly sessions, using non-identical film clips as stimuli. We hypothesized that physiological responses would show high consistency in both the baseline and emotional phases. However, intra- and interindividual variations in the baseline level were relatively large. We demonstrated excellent internal reliability and intraclass consistency in SCL, meanHR, and meanBVP during the emotion-provoking phase throughout the 10 sessions, suggesting that these features can be used as reliable physiological indices in emotion studies. Additionally, our findings can be applied to various emotion-related research fields, such as emotion recognition, development of emotion theory, and profiling emotion-specific physiological responses.

## Supplementary information

**Additional file 1: Table S1**. Physiological responses and their reliability indices from the baseline and emotion-provoking phases of the happiness condition. **Table S2**. Physiological responses and their reliability indices from the baseline and emotion-provoking phases of the sadness condition. **Table S3**. Physiological responses and their reliability indices from the baseline and emotion-provoking phases of the anger condition. **Table S4**. Physiological responses and their reliability indices from the baseline and emotion-provoking phases of the fear condition. **Table S5**. Physiological responses and their reliability indices from the baseline and emotion-provoking phases of the disgust condition. **Table S6**. Physiological responses and their reliability indices from the baseline and emotion-provoking phases of the surprise condition.

## Data Availability

Not applicable

## Abbreviations

ANS: Autonomic nervous system
BVP: Blood volume pulse
ECG: Electrocardiogram
EDA: Electrodermal activity
FT: Fingertip temperature
HR: Heart rate
ICC: Intraclass correlation coefficient
SCL: Skin conductance level
