# Supplementary material for: Reliability of Physiological Responses Induced by Basic Emotions: A Pilot Study
Source: J Physiol Anthropol. 2019 Nov 28;38:15. doi: 10.1186/s40101-019-0209-y (PMC6883530; doi:10.1186/s40101-019-0209-y)
Supplement: Supplementary file 1 — Additional file 1: Table S1. Physiological responses and their reliability indices from the baseline and emotion-provoking phases of the happiness condition. Table S2. Physiological responses and their reliability indices from the baseline and emotion-provoking phases of the sadness condition. Table S3. Physiological responses and their reliability indices from the baseline and emotion-provoking phases of the anger condition. Table S4. Physiological responses and their reliability indices from the baseline and emotion-provoking phases of the fear condition. Table S5. Physiological responses and their reliability indices from the baseline and emotion-provoking phases of the disgust condition. Table S6. Physiological responses and their reliability indices from the baseline and emotion-provoking phases of the surprise condition. [file 40101_2019_209_MOESM1_ESM.pdf]

Table S1. Physiological responses and their reliability indices from the baseline and emotion-provoking phases of the happiness condition

Session	SCL (μS)		meanFT (°C)		meanHR (bpm)		meanBVP (V)	
	Baseline	HAP	Baseline	HAP	Baseline	HAP	Baseline	HAP
1	5.1±3.07	5.1±4.74	32.3±0.02	32.4±0.12	72.2±10.34	72.8±11.07	8.4±0.84	8.4±0.86
2	3.5±3.27	3.8± 3.11	32.4±0.21	32.4±0.02	72.9±11.25	70.9±10.10	8.4±0.61	8.3±0.70
3	4.7±2.69	4.5±2.91	32.4± 0.10	32.3±0.20	71.6±8.84	70.2±9.36	8.2±0.81	8.2±0.66
4	3.6±2.01	4.2±2.57	32.3±0.20	32.4±0.01	74.9±9.08	73.1±9.38	8.7±0.93	8.7±0.98
5	3.9± 2.99	4.3±2.81	34.7±0.71	34.7±0.73	72.4±10.68	73.1±11.77	8.8±0.61	8.7±0.66
6	4.4±2.97	4.1±3.61	35.0±0.48	34.9±0.50	76.0±9.58	76.8±12.22	8.2±0.63	8.2±0.57
7	4.4±2.78	3.1±2.68	35.0±0.75	34.9±0.77	76.4±10.36	75.7±9.01	8.3±0.61	8.2±0.67
8	4.7±3.13	3.9±3.88	35.2±0.56	35.2±0.57	74.4±10.51	72.7±11.89	8.1±0.71	8.6±0.74
9	4.1±2.85	3.0±2.02	35.2±0.38	35.3±0.33	79.1±11.78	76.5±16.09	8.2±0.64	8.5±0.67
10	4.4±2.81	3.3±3.73	35.2±0.68	35.3±0.87	80.0±14.37	78.6±13.24	8.4±0.59	8.8±0.65
Mean	4.3±2.86	3.9± 3.21	34.0±0.36	33.9±0.38	74.9±10.68	74.1±11.72	8.4±0.70	8.5±0.72
α	.61	.95	.69	.69	.44	.92	.63	.95
ρ	.59.	.95	.69	.69	.38	.91	.67	.95

Abbreviations: HAP, happiness; α, Cronbach's alpha; *ρ*, intra-class correlation.

Table S2. Physiological responses and their reliability indices from the baseline and emotion-provoking phases of the sadness condition

Session	SCL (μS)		meanFT (°C)		meanHR (bpm)		meanBVP (V)	
	Baseline	SAD	Baseline	SAD	Baseline	SAD	Baseline	SAD
1	5.0±2.87	5.5±4.42	32.3±0.01	32.4±0.02	70.5±10.15	70.8±10.20	8.5±0.91	8.4±0.92
2	3.9±2.48	3.3±2.50	32.4±0.02	32.4±0.04	69.2±8.57	68.1±8.32	8.1±0.85	8.1±0.80
3	4.7±2.62	3.8±2.42	32.5±0.04	32.3±0.01	69.4±7.78	68.9±8.74	8.1±0.80	8.1±0.84
4	4.1±2.03	3.6±2.15	32.4±0.03	32.9±0.04	73.3±9.84	74.5±11.83	8.5±0.87	8.5±0.85
5	4.5±2.46	4.2±2.89	34.2±1.81	34.4±1.92	72.9±10.07	71.7±11.61	8.6±0.91	8.6±0.98
6	4.4±3.21	4.4±2.68	35.1±0.57	35.1±0.55	73.1±9.56	73.3±10.57	8.2±0.76	8.3±0.70
7	4.3±2.55	4.3±3.20	34.9±0.82	34.9±1.02	75.2±9.57	71.1±10.07	7.9±0.75	8.1±0.87
8	4.2±2.89	4.2±2.97	35.3±0.50	35.2±0.62	74.6±10.31	70.0±9.98	8.3±0.75	8.5±0.94
9	4.5±2.56	3.7±2.05	35.3±0.63	35.3±0.58	75.1±10.77	71.7±11.32	8.0±0.73	8.3±0.91
10	3.9±3.41	3.8±2.84	35.1±0.73	35.2±0.83	76.9±12.14	75.5±11.15	8.2±0.89	8.6±0.99
Mean	4.4±2.71	4.1±2.79	33.9±0.51	34.0±0.56	73.0±10.18	71.6±10.88	8.3±0.83	8.3±0.88
α	.63	.95	.36	.54	.48	.96	.57	.96
ρ	.54	.94	.35	.54	.44	.96	.63	.96

Abbreviations: SAD, sadness; α, Cronbach's alpha; ρ, intra-class correlation.

Table S3. Physiological responses and their reliability indices from the baseline and emotion-provoking phases of the anger condition

Session	SCL (μS)		meanFT (°C)		meanHR (bpm)		meanBVP (V)	
	Baseline	ANG	Baseline	ANG	Baseline	ANG	Baseline	ANG
1	5.2±3.36	4.9±3.00	32.4±0.02	32.3±0.10	71.5±9.45	71.0±11.13	8.4±0.91	8.4±0.90
2	4.1±2.42	3.6±2.33	32.3±0.13	32.4±0.21	71.4±9.31	68.6±10.47	8.1±0.78	8.2±0.71
3	4.6±2.63	4.3±3.11	32.9±0.30	32.4±0.03	71.0±8.91	68.1±9.99	8.1±0.79	8.3±0.78
4	3.9±2.21	3.8±2.69	32.8±0.03	32.4±0.02	73.1±9.62	71.8±10.33	8.6±0.94	8.6±0.95
5	4.3±2.39	4.5±3.15	34.6±0.91	34.7±0.92	70.8±11.11	68.6±9.93	8.6±0.92	8.6±0.95
6	4.2±3.43	3.9±2.73	35.1±0.86	35.0±0.81	74.3±9.06	74.7±11.42	8.6±0.92	8.2±0.60
7	4.6±3.39	4.1±3.12	34.9±0.69	34.9±0.65	74.1±10.23	71.3±8.94	8.3±0.81	8.1±0.86
8	3.6±2.54	4.8±3.19	35.0±0.54	35.1±0.55	73.1±11.06	70.9±10.43	8.3±0.80	8.5±0.90
9	3.7±3.03	3.3±2.18	35.0±1.04	35.1±1.02	75.5±11.79	73.5±12.18	8.3±0.81	8.3±0.79
10	4.2±2.68	4.2±3.31	35.2±0.49	35.2±0.64	78.2±14.77	75.3±13.70	8.3±0.72	8.6±0.87
Mean	4.2±2.81	4.1±2.88	33.9±0.46	34.0±0.47	73.3±10.53	71.4±11.05	8.3±0.84	8.4±0.83
α	.47	.96	.57	.61	.26	.96	.41	.96
ρ	.40	.96	.56	.60	.10	.95	.45	.96

Abbreviations: ANG, anger; α, Cronbach's alpha; ρ, intra-class correlation.

Table S4. Physiological responses and their reliability indices from the baseline and emotion-provoking phases of the fear condition

Session	SCL (μS)		meanFT (°C)		meanHR (bpm)		meanBVP (V)	
	Baseline	FEA	Baseline	FEA	Baseline	FEA	Baseline	FEA
1	4.8±2.88	5.9±3.75	32.4±0.12	32.3±0.11	69.8±10.28	70.1±10.79	8.4±0.90	8.3±0.92
2	3.8±2.78	4.2±3.04	32.9±0.22	32.3±0.12	69.2±8.83	68.9±8.17	8.2±0.89	8.1±0.88
3	4.4±2.88	5.7±2.68	32.4±0.13	32.3±0.03	70.9±6.99	70.0±9.22	8.1±0.89	8.1±0.82
4	4.1±2.14	4.7±1.58	32.8±0.22	32.3±0.25	73.3±10.48	73.1±11.94	8.6±0.86	8.5±0.89
5	4.6±2.60	5.3±2.85	34.6±0.95	34.6±1.03	71.5±12.06	68.6±10.71	8.6±0.96	8.5±0.97
6	4.7±3.18	5.8±2.36	35.1±0.97	35.1±1.00	75.3±10.79	72.9±10.55	8.5±0.89	8.2±0.69
7	4.9±3.00	4.5±2.73	35.0±0.68	34.9±0.68	73.6±9.79	74.7±9.87	8.6±0.93	8.1±0.74
8	4.7±3.15	7.3±2.75	35.3±0.70	35.2±0.62	74.3±11.12	69.9±12.30	8.7±0.96	8.5±0.98
9	5.0±2.99	5.3±2.15	35.1±0.82	35.0±0.75	78.3±10.75	74.8±12.28	8.5±0.92	8.2±0.93
10	4.9±2.39	5.1±2.93	35.5±0.67	35.4±0.68	76.9±13.52	75.5±12.86	8.5±0.90	8.7±0.82
Mean	4.6±2.80	5.4±2.68	34.0±0.49	34.0±0.48	73.3±10.46	71.9±10.87	8.5±0.92	8.3±0.86
α	.34	.91	.52	.62	.50	.94	.56	.96
ρ	.30	.91	.52	.61	.40	.94	.56	.96

Abbreviations: FEA, fear; α, Cronbach's alpha; ρ, intra-class correlation.

Table S5. Physiological responses and their reliability indices from the baseline and emotion-provoking phases of the disgust condition

Session	SCL (μS)		meanFT (°C)		meanHR (bpm)		meanBVP (V)	
	Baseline	DIS	Baseline	DIS	Baseline	DIS	Baseline	DIS
1	5.1±3.11	5.6±4.06	32.9±0.21	32.8±0.11	72.8±10.09	70.8±12.05	8.4±0.90	8.3±0.90
2	3.9±2.75	4.8±3.66	32.8±0.11	32.7±0.12	70.5±7.86	69.3±9.55	8.3±0.77	8.2±0.74
3	4.6±2.86	4.8±3.07	32.8±0.31	32.8±0.21	71.6±7.52	71.6±8.77	8.2±0.64	8.2±0.64
4	3.9±2.00	4.6±2.37	32.9±0.12	32.8±0.22	73.8±10.17	71.9±10.39	8.6±0.87	8.5±0.85
5	4.5±2.27	5.1±2.74	34.5±0.94	34.4±0.96	71.8±10.65	67.2±11.02	8.6±0.86	8.6±0.90
6	3.7±2.59	5.4±3.12	35.1±0.92	35.1±0.96	75.3±11.05	73.5±9.46	8.5±0.89	8.2±0.69
7	3.4±2.19	4.7±3.13	34.9±0.83	34.8±0.68	75.9±10.11	73.9±9.19	8.4±0.64	8.3±0.85
8	3.9±2.64	5.8±3.43	35.2±0.59	35.1±0.70	73.3±10.27	68.9±9.98	8.6±0.81	8.5±0.82
9	4.1±2.51	4.2±2.25	35.2±0.42	35.1±0.50	78.1±6.74	75.3±12.91	8.4±0.94	8.2±0.87
10	3.8±2.83	4.1±2.04	35.2±0.45	35.3±0.59	77.6±12.94	73.6±12.04	8.5±0.94	8.5±0.93
Mean	4.1±2.58	4.9±2.99	34.0±0.42	33.9±0.45	74.1±9.74	71.6±10.54	8.4±0.84	8.3±0.82
α	.57	.94	.13	.39	.46	.96	.71	.96
ρ	.57	.93	.13	.38	.40	.96	.70	.96

Abbreviations: DIS, disgust; α, Cronbach's alpha; ρ, intra-class correlation.

Table S6. Physiological responses and their reliability indices from the baseline and emotion-provoking phases of the surprise condition

Session	SCL (μS)		meanFT (°C)		meanHR (bpm)		meanBVP (V)	
	Baseline	SUR	Baseline	SUR	Baseline	SUR	Baseline	SUR
1	5.1±3.45	6.5±3.11	32.4±0.01	32.3±0.12	71.1±11.06	70.5±10.52	8.3±0.86	8.3±0.95
2	4.0±2.85	4.6±2.26	32.4±0.12	32.4±0.02	72.9±9.13	69.7±8.49	8.3±0.83	8.2±0.74
3	3.8±3.39	3.6±3.11	32.8±0.20	32.4±0.11	72.0±7.19	69.8±8.17	8.3±0.71	8.4±0.59
4	3.9±3.03	4.8±1.98	32.9±0.12	32.8±0.22	73.7±8.71	73.8±10.51	8.7±0.86	8.5±0.87
5	4.1±2.21	4.1±2.35	34.4±1.0	34.5±1.15	72.4±13.69	68.1±10.48	8.7±0.81	8.6±0.87
6	4.2±2.90	5.8±3.01	35.3±0.61	35.2±0.50	74.7±10.90	72.3±10.94	8.5±0.86	8.2±0.71
7	3.6±1.55	4.6±3.09	34.9±0.72	34.9±0.76	75.6±11.40	74.2±10.11	8.6±0.93	8.1±0.75
8	3.9±2.38	5.9±3.02	35.3±0.75	35.2±0.66	75.9±11.75	71.0±12.19	8.4±0.98	8.5±1.00
9	4.5±3.14	4.7±2.52	35.4±0.40	35.3±0.39	73.7±10.91	72.5±10.93	8.5±0.94	8.3±0.98
10	3.9±2.50	4.7±2.45	35.3±0.38	35.4±0.56	77.8±11.84	75.4±14.23	8.4±0.89	8.5±0.96
Mean	4.1±2.74	4.9±2.89	34.1±0.40	34.0±0.41	74.0±10.66	71.7±10.66	8.5±0.88	8.3±0.84
α	.79	.92	.63	.72	.54	.96	.71	.96
ρ	.45	.92	.63	.72	.47	.95	.70	.96

Abbreviations: SUR, surprise; α, Cronbach's alpha; ρ, intra-class correlation.
